# The Pharmacoeconomic Impact of Pharmaceutical Care in the Hospital: Protocol for an Overview of Systematic Reviews

**DOI:** 10.2196/35865

**Published:** 2023-04-21

**Authors:** Fábio Jorge Ramalho de Amorim, Fernanda Valença-Feitosa, Marcos Cardoso Rios, Carlos Adriano Santos Souza, Izadora Menezes da Cunha Barros, Alfredo Dias de Oliveira-Filho, Divaldo Pereira de Lyra-Júnior

**Affiliations:** 1 Pharmacy Department Laboratory of Teaching and Research in Social Pharmacy Federal University of Sergipe São Cristóvão Brazil; 2 University Center AGES Paripiranga Brazil; 3 Pharmacy Department Federal University of Sergipe, Campus Lagarto Lagarto Brazil

**Keywords:** pharmacoeconomics, pharmaceutical care, hospital, overview, cost-effectiveness, cost benefit, cost-utility

## Abstract

**Background:**

The clinical activities developed by pharmacists in a hospital environment can improve health outcomes and generate savings for hospitals. However, to determine whether pharmaceutical interventions are cost effective, it is essential to define a method according to which cost-effectiveness is intended to be measured. In addition, the quality of economic assessments and the amount of information present in systematic reviews in the literature make it difficult to analyze the effects of this intervention.

**Objective:**

This paper aims to provide an overview of systematic reviews on the pharmacoeconomic impact of the performance of pharmaceutical care in hospitals.

**Methods:**

A systematic search of the Cochrane Library databases, PubMed or MEDLINE, LILACS, Scopus, Web of Science, Google Scholar, and Open Thesis will be performed using the PRISMA (Preferred Reporting Items for Systematic Reviews and Meta-Analyses) statement. The search will involve the use of keywords determined using the Medical Subject Headings database to define the search terms and include the following terms: “pharmacoeconomics,” “pharmaceutical care,” and “hospital.” The study designs to be included will be systematic reviews of good quality. Studies will be included that address pharmacoeconomics; studies that evaluated pharmaceutical care in hospitals; and studies published in Portuguese, English, or Spanish. The primary outcome sought in the systematic reviews will be the cost ratio in monetary units and the outcomes in monetary or natural units. The secondary economic outcomes considered will be determined based on factors associated with the drugs and translated into benefit, efficacy, or utility.

**Results:**

It is intended to start this overview in January 2023. Thus far, only previous searches have been carried out to contextualize the theme and build the protocol.

**Conclusions:**

This overview will determine the pharmacoeconomic impact of pharmaceutical care interventions in the hospital environment. In addition, this study will point out which clinical outcomes in natural units are impacted by the performance of pharmaceutical care and the strengths and limitations of each approach. It will also identify gaps in the literature and areas for future work.

**Trial Registration:**

PROSPERO CRD42019140665; https://tinyurl.com/bddwnz43

## Introduction

Within the scope of pharmacy, clinical pharmaceutical services promoted actions that improve clinical and economic outcomes [1-3]. In clinical practice, pharmaceutical activities focused on patient care are performed in close cooperation with the health team to offer the best patient care. Clinical activities developed by these pharmacists in a hospital environment can minimize medication errors, improve the results of pharmacotherapy, and decrease treatment costs [4-6]. In this sense, research that describes and economically evaluates the provision of pharmaceutical care in hospitals has been identified in the literature since the 1970s [7].

However, to determine whether pharmaceutical interventions are cost effective, it is essential to define a method according to which the intervention is intended to be measured, taking into account the limitations of each step and the potential for generalizing the results to assist in the decision-making process [7,8]. There are 4 basic types of studies related to this issue, which are cost-minimization, cost-benefit, cost-effectiveness, and cost-utility analyses. These analyses differ fundamentally from each other in terms of how health outcomes are measured and compared [1,7].

Pharmacoeconomics has, therefore, emerged as a tool to optimize the use of financial resources for health care without prejudice about the quality of treatment, to reconcile therapeutic needs with the costing possibilities for decision-making, having a crucial role in clinical decisions, management pharmacotherapy, and drug use guidelines [2,9].

It is worth mentioning that several studies, systematic reviews, and meta-analyses have shown that pharmaceutical care generates savings for hospitals and health systems, and pharmaceutical care is one of the strategies to improve patient health outcomes [9-12]. However, there is no information synthesis of the existing information about this service, making it difficult to take the data resources used [13]. Thus, this study is aimed at providing an overview of systematic reviews on the pharmacoeconomic impact of the performance of pharmaceutical care in hospitals [14-16].

## Methods

### Overview

This study protocol is reported according to the reporting guidelines provided in the Statement of Preferred Reporting Items for Systematic Reviews and Meta-Analysis Protocols (PRISMA-P) [17]. The review protocol was registered in PROSPERO (CRD42019140665).

### Information Sources and Search Strategy

A systematic search of the Cochrane database, PubMed, Medline, LILAC, Scopus, Web of Science, Google Scholar, and Open Thesis will be conducted. The search strategy will use terms from the Medical Subject Headings and Descriptores en Ciencias de la Salud databases, as follows: (pharmacoeconomics); (‘Pharmaceutical economics’) (‘pharmacy economics’); (‘Pharmacy economic’); (‘Pharmaceutical economics’); and (‘Clinical pharmacy services’), (‘clinical pharmacy service’), (‘pharmaceutical care’), (‘pharmacy service, hospital’), (hospital), (hospitals). See Textbox 1 for a detailed search strategy.

**Search strategy—PubMed or Medline (no time limit).**

((“economics, pharmaceutical”[MeSH Terms] OR (“economics”[All Fields] AND “pharmaceutical”[All Fields]) OR “pharmaceutical economics”[All Fields] OR “pharmacoeconomics”[All Fields]) OR (“economics, pharmaceutical”[MeSH Terms] OR (“economics”[All Fields] AND “pharmaceutical”[All Fields]) OR “pharmaceutical economics”[All Fields] OR (“pharmaceutical”[All Fields] AND “economics”[All Fields])) OR (“economics, pharmaceutical”[MeSH Terms] OR (“economics”[All Fields] AND “pharmaceutical”[All Fields]) OR “pharmaceutical economics”[All Fields] OR (“pharmacy”[All Fields] AND “economics”[All Fields]) OR “pharmacy economics”[All Fields]) OR (“economics, pharmaceutical”[MeSH Terms] OR (“economics”[All Fields] AND “pharmaceutical”[All Fields]) OR “pharmaceutical economics”[All Fields] OR (“pharmacy”[All Fields] AND “economic”[All Fields]) OR “pharmacy economic”[All Fields]) OR “economics, pharmaceutical”[mesh]) AND ((“pharmacy service, hospital”[MeSH Terms] OR (“pharmacy”[All Fields] AND “service”[All Fields] AND “hospital”[All Fields]) OR “hospital pharmacy service”[All Fields] OR (“clinical”[All Fields] AND “pharmacy”[All Fields] AND “services”[All Fields]) OR “clinical pharmacy services”[All Fields]) OR (“pharmacy service, hospital”[MeSH Terms] OR (“pharmacy”[All Fields] AND “service”[All Fields] AND “hospital”[All Fields]) OR “hospital pharmacy service”[All Fields] OR (“clinical”[All Fields] AND “pharmacy”[All Fields] AND “service”[All Fields]) OR “clinical pharmacy service”[All Fields]) OR “pharmacy service, hospital”[mesh]) AND ((“hospitals”[MeSH Terms] OR “hospitals”[All Fields] OR “hospital”[All Fields]) OR “hospitals”[mesh])

### Inclusion Criteria

The eligibility criteria were defined according to the PICO strategy (that is, population, intervention, comparison, and result) with the following variables: (1) pharmacoeconomic studies (cost-minimization, cost-benefit, cost-effectiveness, and cost-utility analyses), (2) studies that evaluated pharmaceutical care in hospitals, and (3) studies published in Portuguese, English, or Spanish.

### Exclusion Criteria

Systematic reviews in which the interventions performed by pharmacists in the health team are not distinguishable—(1) overviews; (2) systematic reviews that analyze nonclinical activities, such as medication handling, storage, administration (including vaccines); or (3) other logistical activities—will be excluded.

### Study Design

This study will provide an overview of systematic reviews. The overview seeks to meet the need for evaluating and synthesizing the results of systematic reviews. In addition to optimizing and improving access to information and decision-making, assisting in the synthesis of information for health professionals, managers, researchers, and patients is also crucial [18]. Thus, the following publications obtained from a systematic search of more than one database of literature will be considered: publications that specify the review question, eligibility criteria, and selection of the studies involved; in which data collection was performed by two or more reviewers; in which the risk of bias in the included studies was evaluated; and in which information is synthesized using a quantitative or qualitative approach.

### Population of the Included Studies

Studies that included hospitalized patients who were using at least one medication will be considered.

### Scenarios of the Included Studies

Studies conducted in hospitals anywhere in the world will be considered as long as they meet the inclusion criteria.

### Intervention or Comparison

Studies comparing the clinical results of patients receiving pharmaceutical care services to those of patients for whom there were no pharmacist interventions will be considered.

### Outcomes of Interest

The primary result will be the ratio of cost (currency unit) to the outcome (currency unit or natural units, for example, years of life or quality-adjusted life-years). This is usually presented as an outcome. The secondary economic results will be the determinants or factors associated with medications translated into benefits (such as days of hospitalization avoided; days of work that will be lost; and materials, labor, and equipment that could be relocated), effectiveness (in terms of parameters such as years of life gained, lives saved, cholesterol reduction, mmHg of reduced blood pressure, number of cases prevented, time of symptoms, and reduction of recurrence rates), and usefulness (quality-adjusted life-years).

### Screening and Selection of Studies

All articles identified in the literature search will be screened by 2 reviewers, regardless of duplicate removal using EndNote (Clarivate Plc). The titles and abstracts of the articles returned from the initial searches will be selected based on the eligibility criteria described above (“Inclusion Criteria”). The full texts will be examined in detail and selected for eligibility. The references of all articles considered will be searched manually to identify any relevant reports lost in the search strategy. Any disagreements will be resolved by discussion, if necessary. A PRISMA flowchart showing details of the included and excluded studies at each stage of the study selection process will be provided.

### Data Extraction and Management

A data extraction form will be designed using Google Forms. Google Forms will allow the management of articles and allow authors to collaborate simultaneously. The review team will receive training on how to use Google Forms before the start of the study to ensure the calibration of the forms and methods of data collection. The data to be collected in this overview will be conducted via the items described in Table 1.

**Table 1:** Elements for data collection.

Aspects of data collection	Description of key items to be extracted
Research site characteristics of the studies included in the review	Review objectives Numbers of studies and patients included Period chosen to search for studies Eligibility criteria Profile of the patients included in the studies Diagnostics or comorbidities of the patients studied
Hospital characteristics of the studies included in the review	Size of the hospital characterized by the number of beds and employees Geographic locations of the primary studies Any other relevant resources presented in each review about the primary studies
The pharmacoeconomic evaluation method used	Economic: Cost-benefit Cost-effectiveness Cost minimization Cost utility Humanistic: Quality of life Patient preferences Patient satisfaction
Results of clinical services	Reduced hospital stay Hospital readmission Decrease in adverse drug events Types of pharmaceutical care Improvement or worsening of the clinical parameters evaluated

### Study Characteristics

The following data will be extracted from the studies selected: name of the first author, study design, year of publication, journal, year (or period) of the study, sample size, scenario, and geographic location of the study, among others. Data relevant to the evaluation of the methodological quality of the studies will be collected.

### Methodological Quality Assessment

The evaluation of the methodological quality of systematic reviews will be performed using the Revised Assessment of Multiple Systematic Reviews (R-AMSTAR) instrument. R-AMSTAR is a revised version of AMSTAR, which consists of 11 items with good content validity for measuring the methodological quality of systematic reviews. It is widely accepted and used because of its reliability and reproducibility. Before the start of the assessment, the items that make up the list will be widely discussed, and a manual will be created to guide the interpretation of the R-AMSTAR items to ensure consistency in the analysis.

For the evaluation of systematic reviews, the following items will be considered: conducting a project; the selection of studies and data extraction search in pairs; the scope of the bibliographic research; the analysis of the type of publication (for example, theses, dissertations, and book chapters); the availability of the included and excluded studies; providing the characteristics of the included studies, evaluation, and documentation of the scientific quality of the included studies; the use of the scientific quality of the studies included in the formulation of the conclusions; the suitability of the methods used to combine the findings of the studies; the assessment of the likelihood of publication bias; and the inclusion of conflicts of interest.

### Summary of the Data

Data from each study will be used to create tables of evidence for a general description of the included studies. Monetary values will be presented as mean and standard deviation, and natural outcomes will be presented by descriptive methods. We will also establish a model of quality effects to examine how the quality of each study changes the average result. A comparison of the review methods will be performed in terms of the eligibility criteria (ie, criteria used to identify eligible patients, study designs, and end points of interest), details of bibliographic search (ie, dates, databases, main differences in the strategies employed, and taking into account language restrictions), end point definitions used, and rigorous review methods (as reflected by variations in R-AMSTAR assessments and other aspects of the study methodology). The statistical program SPSS version 17.0 (IBM Corp) will be used to calculate the kappa index to verify the agreement in the selection of studies included among the authors, thus decreasing the chance of missing out on a study and the possibility of bias. In addition to the above, the Risk of Bias in Systematic Reviews tool will be used, which assesses both the risk of bias and the relevance of the research question to be answered [19].

### Ethics Approval and Dissemination

The review will be performed under “Master's research” approved by the Ethics Committee of the University Hospital of the Federal University of Sergipe (CAAE: 66439017.3.0000.5546; seem: 2.300.170). The results will be submitted to indexed journals. We will present at national and international meetings.

## Results

It is intended to start this protocol in January 2023. Thus far, only previous research has been carried out to contextualize the topic and build the protocol. Our objective is to understand the economic impact of pharmaceutical care performance in hospitals, especially about the ratio between cost (economic unit) and outcome (economic or natural units). We also hope to achieve the following: understand the determinants or factors associated with drugs translated into tools, effort, and utility; support health care policy decision makers to improve the optimization of the treatment of these patients so that it can be replicated in other settings of the health care system; and increase the safety and lives of the patients.

## Discussion

This protocol will determine the pharmacoeconomic impact of pharmaceutical care interventions in the hospital environment. In addition, this study will point out which clinical outcomes in natural units are impacted by the performance of pharmaceutical care. Although studies have shown the positive clinical, economic, and human benefits of the interventions of pharmaceutical care for patients and institutions, the subject is not yet exhausted, and the analysis of robust scientific evidence can improve the significance of the results. This study will identify and fill the knowledge gaps in the field [20].

According to De Rijdt et al [21], the economic evaluations of clinical pharmacy services, for the most part, have a series of methodological limitations related to the absence of a control group without pharmaceutical care interventions, the limited scope of costs and results, focus being limited to direct health costs, the exclusion of the cost of employing pharmacists, the use of intermediate outcomes, the exclusion of health benefits, and the absence of incremental cost analysis.

In their assessment of pharmaceutical care, Touchette et al [20] reported that most studies were conducted in hospitals with the most common types of pharmacotherapeutic monitoring and disease management services. The authors highlight the variation in the quality of studies, with less than half being considered good or reasonable [19]. In this sense, it is necessary to better understand the effects of these pharmaceutical interventions, which makes it possible to define strategies to facilitate their implementation and integration in hospital care. Consequently, it is possible to overcome their negative effects and optimize the positive effects to use them to their full potential as a tool to support pharmaceutical care and ultimately improve the results [22].

Any changes made to this protocol during the course of the study will be reported in the final manuscript and reported in PROSPERO. The results will be made public through publication in a peer-reviewed journal. There are several limitations to our planned systematic review. We plan to identify reviews that are indexed in specific databases, which may restrict the inclusion of systematic reviews that are not in the selected databases. Furthermore, there is a limitation related to the language, since the search for manuscripts will be performed in only 3 languages (Portuguese, English, and Spanish).

## Abbreviations

PRISMA-P: Preferred Reporting Items for Systematic Reviews and Meta-Analysis Protocols
R-AMSTAR: Revised Assessment of Multiple Systematic Reviews

## Data Availability

Data sharing is not applicable to this article as no data sets were generated or analyzed during this study.
